# Promising score for teaching and learning environment: an experience of a fledgling medical college in Saudi Arabia

**DOI:** 10.1186/s12909-023-04357-3

**Published:** 2023-06-27

**Authors:** Mohammed Almansour, Bader A. AlMehmadi, Nida Gulzar Zeb, Ghassan Matbouly, Waqas Sami, Al-Mamoon Badahdah

**Affiliations:** 1 Department of Medical Education, College of Medicine, King Saud University, Riyadh, 11461 Saudi Arabia; 2 Department of Internal Medicine, College of Medicine, Majmaah University, Al Majmaah, 15341 Saudi Arabia; 3 Department of Basic Medical Sciences, College of Medicine, Majmaah University, Al Majmaah, 15341 Saudi Arabia; 4 Department of Medical Education, College of Medicine, Majmaah University, Al Majmaah, 15341 Saudi Arabia; 5 College of Nursing, QU Health, Qatar University, P.O Box 2713, Doha, Qatar; 6 Department of Family and Community Medicine, Faculty of Medicine in Rabigh, King Abdulaziz University, Jeddah, 22252 Saudi Arabia

**Keywords:** ETLQ, Teaching & learning, Education environment, Students’ perception, Integrated curriculum, Outcome-based curriculum, Fledgling college

## Abstract

**Background:**

Professional competency of graduates of an institute reflects its teaching and learning environment (TLE). This study aimed to provide a preliminary assessment of the TLE at the College of Medicine at Majmaah University.

**Methods:**

A cross-sectional survey was conducted during the 2019-20 academic year among students at the College. A validated scoring tool “the Experience of Teaching and Learning Questionnaire” (available at https://bit.ly/3sVBuEw) was used. The mean score of each section and statement, the difference between the mean scores of different demographic groups, and correlations between sections were analysed.

**Results:**

A total of 234 (72.2%) enrolled students participated in this survey, with a male-to-female ratio and a ratio of participants from basic to clinical years being 2:1 and 1:1, respectively. Most participants reported a GPA of above 3/5. The overall mean score was 3.52/5 points. Section one “approaches to learning and studying” has the highest mean score (3.68), and no section scored a mean below three, though section three “demands made by the course” scored a borderline mean of 3.08. Students in clinical years had a significantly higher overall mean score compared to their counterparts (3.66 vs. 3.39, *p* < 0.001).

**Conclusions:**

Students at the College had a positive perception of the TLE, but face challenges in coping with the demands of acquiring knowledge and subject-based skills, and in appreciating the TLE especially during basic science years, highlighting the need for an atmosphere that allows them to meet demands and develop greater appreciation.

## Introduction

The professional competency of graduates reflects the pedagogical, physical, and psychosocial context of the institutes they have attended, known as the teaching and learning environment (TLE). It is the atmosphere in which students work and learn [1–4]. Positive TLE is essential for a successful institutional educational program and student professional and moral development [5, 6], hence, the development of a competent graduate [2, 7, 8]. Both the learning and academic well-being of students improve noticeably when the TLE is supportive [9–11]. Non-academic aspects such as feeling, mood, and burnout are remarkably affected by a non-supportive environment [12].

Accreditors and professional organizations indicate the necessity of periodic evaluation and assessment of the TLE [10, 13]. Monitoring how students perceive the TLE is an effective method to evaluate and improve it [14, 15]. Understanding the perception of students is important in order to evaluate and transform the educational program and provide insight to enhance the learning environment [14–16]. The better the students perceive their TLE, the better their overall performance was [17]. Reviewers have identified 28 unique assessment tools developed to measure how medical students and residents perceive the TLE in their schools [18].

A large multi-institutional cohort study found that establishing learning communities at medical schools was associated with more positive perceptions of the learning environment by pre-clerkship students [19]. A few factors which influence the students’ perception of the learning environment the most are the quality of teaching and faculty, assessment methods, curriculum design, and free spaces for self-directed learning [20]. A study in Saudi Arabia on undergraduate medical students’ preferred learning styles revealed that further research is needed before claiming that the relationship between learning style preferences and teaching and learning tactics is better understood [21].

The College of Medicine in Majmaah is an evolving educational institution established in 2009 in a small city with relatively limited medical infrastructure. National and international medical education experts contribute to constructing the college’s own integrated outcome-based hybrid curriculum. Students must complete a preparatory year before joining the five-year module-based medical program, followed by a one-year mandatory internship. The college accommodates 324 students and has five batches of graduates.

Despite numerous existing studies evaluating the TLE in Saudi Arabia, a gap persists in the assessment of newly established medical colleges, which often have unique themes and curricula. This highlights the need for further investigation into the TLE in these specific contexts.

To this end, this study aims to provide a preliminary assessment of the learning environment at the College of Medicine at Majmaah University from the students’ perspective. The primary objective is to evaluate the students’ perceptions of the TLE at the college. Subsequently, the study seeks to (a) explore the difference in perception among key demographic groups, and (b) identify areas for improvements.

## Methods

This was an observational, cross-sectional survey conducted during the 2019-20 academic year among students at the College of Medicine at Majmaah University. All enrolled students received an e-mail message from the Office of Students Affairs, inviting them to participate in the survey with a link to complete a web-based questionnaire. Additionally, batch leaders sent frequent reminders to encourage student participation, and the research team effectively communicated with students through regular follow-up messages and reminders. Potential participants were initially offered a brief introduction to the study and given the option to confirm their willingness to participate before being directed to the e-questionnaire. The data was collected using Google Form™ (Alphabet Inc., Mountain View, CA, USA) and subsequently exported to a Microsoft Excel™ (Microsoft™ Corp., Redmond, WA, USA) spreadsheet for analysis.

### Study instrument

An anonymous validated scoring tool, the Experience of Teaching and Learning Questionnaire (ETLQ) [22], was used to assess the students’ perceptions of the TLE. The official long English version of the questionnaire was used, given that all participants were proficient in English. Nevertheless, Arabic descriptions were incorporated within the English version for further clarification.

The ETLQ (available at https://bit.ly/3sVBuEw) is a self-reported assessment tool that consists of five sections. The first section, “learning and studying approaches,“ is composed of 18 statements that assess how students have been studying. The second section, “teaching and learning experiences,“ comprises 40 statements that evaluate students’ perceptions of the TLE. The third section consists of ten statements that assess the “demands made by the course” and how challenging students found different aspects of it. The fourth section contains eight statements that explore what students have learned during the course. The fifth and final section includes one item that assesses how well students believe they performed in the course. Students rated their responses to each statement on a five-point scale.

### Statistical analysis

The data were tested for accuracy and quality before analysis commenced. Empty submissions or those with a similar response for all items were considered invalid participation and were excluded from the study. Submissions with a similar response for all items of a section or with insufficient data (< 60% of section/scale items) were excluded from the analysis of that particular section. Subsequently, the data was imported to the Statistical Package for Social Sciences (SPSS™) for Windows™ v.25.0 (IBM Corp., Armonk, NY, USA) for analysis. Responses were coded on a scale of 1 to 5 following the ETLQ scoring key (available at www.etl.tla.ed.ac.uk/questionnaires/scoringkey.pdf).

The mean score and standard error of mean (SEM) for each participant for each section were calculated before analysing the overall mean score and SEM at the sections level. A t-test was applied to evaluate the difference between the means of males and females, and participants from basic science and clinical years. Additionally, the mean score and SEM for each statement of the ETLQ were analysed. Pearson correlation was applied to observe correlations between sections. A *p*-value of < 0.05 was considered statistically significant.

This study was ethically approved by the Research Ethics Committee of Majmaah University (Ref: HA-01-R-008, date: 26-03-2020).

## Results

A total of 234 (72.2%) out of 324 students enrolled in the college participated in this survey. Only 228 were included in the analysis (6 submissions were excluded due to empty or invalid responses). The ratio of male-to-female, and participants from basic to clinical years were 2:1 and 1:1 respectively. Most of the sample were enrolled in the second and fourth year (35.5% and 27.2% respectively). The majority (88.4%) of participants have reported a personal grade point average (GPA) above three out of five as seen in Table 1.

**Table 1:** Demographic Characteristics of Participants

Characteristic	All n (%)	Male n (%)	Female n (%)		
Number of participants	228/324* (70.4)	151/233* (64.8)	73/91* (80.2)		
Age (years)					
Mean (SD)	21.78 (± 1.97)	21.9 (± 1.93)	21.6 (± 1.97)		
Gender**					
Male:female	2:1	-	-		
Academic year***					
Basic sciences	116 (50.9)	73 (48.7)	40 (54.8)		
2nd year	81 (35.5)	46 (30.5)	32 (43.8)		
3rd year	35 (15.4)	27 (17.9)	8 (11.0)		
Clinical years	112 (49.1)	78 (51.3)	33 (45.2)		
4th year	62 (27.2)	50 (33.2)	12 (16.4)		
5th year	24 (10.5)	12 (7.9)	12 (16.4)		
6th year	21 (9.2)	12 (7.9)	8 (11.0)		
Internship	5 (2.2)	4 (2.6)	1 (1.4)		
GPA					
< 2	1 (0.4)	1 (0.7)	0 (0)		
2–3	26 (11.2)	18 (11.8)	7 (9.6)		
3–4	73 (33.2)	54 (35.8)	18 (24.6)		
4–5	128 (55.2)	78 (51.7)	48 (65.8)		
Last module taken					
Basic clinical skill	27 (13.9)	22 (17.3)	5 (7.6)		
Principles of health and disease	25 (12.9)	23 (18.1)	2 (3.0)		
Human body	23 (11.9)	15 (11.8)	7 (10.6)		
Internal medicine 1	19 (9.8)	10 (7.9)	9 (13.6)		
GIT	18 (9.3)	14 (11.0)	4 (6.1)		
Family medicine	15 (7.7)	13 (10.2)	2 (3)		
Circulation & breathing	13 (6.7)	12 (9.4)	1 (1.5)		
Multisystem disorder	10 (5.2)	7 (5.5)	3 (4.5)		
Pharmacology	9 (4.6)	0 (0)	9 (13.6)		
Emergency medicine	8 (4.1)	1 (0.8)	7 (10.6)		
Other	27 (13.9)	10 (7.9)	17 (25.8)		

*n/N, total participants to the total number of enrolled students in the college.

**Four participants did not declare their gender.

***First year is an orientation year before joining college of medicine.

The overall mean score for all of the 76 statements was 3.52/5 points, and the standard error of mean (SE) was ± 0.04. There was no difference between students at male and female campuses in the overall mean score (3.56 SE ± 0.04 vs. 3.47 SE ± 0.07 *p* = 0.230), in contrast, students in clinical years have a significantly higher overall mean score compared to their counterparts in basic science years (3.66 SE ± 0.05 vs. 3.39 SE ± 0.06, *p* < 0.001).

At the sections level, Table 2 highlghts the mean score of each section and the mean difference between two key groups, namely male vs. female and students in basic vs. clinical years. Section one “approaches to learning and studying” received the highest score (3.68 SE ± 0.03). No section scored a mean below three, though section three “demands made by the course unit” had a borderline mean of 3.08 ± 0.05. No significant difference in the mean score was observed between male and female students at the section level. In contrast, students in clinical years had a significantly higher mean score compared to those in basic science years in three sections, specifically in sections two (*p* < 0.001), three (*p* = 0.04), and five (*p* = 0.01) (Table 2).

**Table 2:** Overall mean score with comparison among key students’ groups

	Mean score ± standard error of mean						
Sections (n)	**Overall**	**Male**	**Female**	***p-value***	**Basic Science**	**Clinical years**	***p-value***
Overall (228)	3.52 ± 0.04	3.56 ± 0.04	3.47 ± 0.07	0.230	3.39 ± 0.06	3.66 ± 0.05	0.000**
Section 1 (218)	3.68 ± 0.03	3.66 ± 0.49	3.71 ± 0.04	0.445	3.73 ± 0.05	3.62 ± 0.04	0.061
Section 2 (205)	3.57 ± 0.05	3.63 ± 0.06	3.51 ± 0.1	0.259	3.33 ± 0.08	3.82 ± 0.06	0.000**
Section 3 (185)	3.08 ± 0.05	3.13 ± 0.06	2.97 ± 0.08*	0.113	2.98 ± 0.07*	3.17 ± 0.07	0.040**
Section 4 (162)	3.33 ± 0.05	3.28 ± 0.07	3.4 ± 0.07	0.210	3.25 ± 0.07	3.42 ± 0.06	0.078
Section 5 (218)	3.23 ± 0.07	3.17 ± 0.08	3.38 ± 0.13	0.169	3.06 ± 0.1	3.4 ± 0.09	0.010**

Section 1, approaches to learning and studying; Sect. 2, experiences of teaching and learning; Sect. 3, demands made by the course unit; Sect. 4, what you learned from this course unit.

*Mean score below 3 points.

**Statistically significant.

The mean score for each of the 76 statements are shown in Table 3 (for Sects. 1, 3, and 4) and Table 4 (for Sect. 2). Section one “approaches to learning and studying” achieved the highest mean score of 3.68 SE ± 0.03, with the statement “I have generally put a lot of effort into my studying” receiving the overall highest mean score of4.31 ± 0.06. However, two statements (13 and 17) scored a mean below three in this section (Table 3).

**Table 3:** Sections 1, 3, and 4: mean score for each statement

Section 1: Approaches to learning and studying			Section 3: Demands made by the course unit				Section 4: What you learned from this course unit				
#	**Statement**	**Mean score ± SE**	**#**	**Statement**	**Mean score ± SE**	**#**		**Statement**	**Mean score ± SE**		
1.	I’ve often had trouble in making sense of the things I have to remember.	3.5 ± 0.08	a.	What I was expected to know to begin with.	3.1 ± 0.07	a.		Knowledge and understanding about the topics covered.	3.4 ± 0.08		
2.	I’ve been over the work I’ve done to check my reasoning and see that it makes sense	3.97 ± 0.06	b.	The rate at which new material was introduced.	3.02 ± 0.08	b.		Ability to think about ideas or to solve problems.	3.34 ± 0.07		
3.	I have usually set out to understand for myself the meaning of what we had to learn.	4.06 ± 0.06	c.	The ideas and problems I had to deal with.	2.81 ± 0.07*	c.		Skills or technical procedures specific to the subject.	3.2 ± 0.08		
4.	I have generally put a lot of effort into my studying.	4.31 ± 0.06	d.	The skills or technical procedures needed in this subject.	3.01 ± 0.07	d.		Ability to work with other students.	3.3 ± 0.08		
5.	Much of what I’ve learned seems no more than lots of unrelated bits and pieces in my mind	3.07 ± 0.08	e.	The amount of work I was expected to do.	2.74 ± 0.07*	e.		Organising and being responsible for my own learning.	3.47 ± 0.08		
6.	In making sense of new ideas, I have often related them to practical or real-life contexts	4.02 ± 0.06	f.	Working with other students.	3.38 ± 0.08	f.		Ability to communicate knowledge and ideas effectively.	3.24 ± 0.07		
7.	On the whole, I’ve been quite systematic and organised in my studying.	3.43 ± 0.08	g.	Organising and being responsible for my own learning.	3.06 ± 0.07	g.		Ability to track down information in this subject area.	3.34 ± 0.07		
8.	Ideas I’ve come across in my academic reading often set me off on long chains of thought.	3.84 ± 0.06	h.	Communicating knowledge and ideas effectively.	3.02 ± 0.07	h.		Information technology/computing skills (e.g., WWW, email, word processing).	3.41 ± 0.08		
9.	I’ve looked at evidence carefully to reach my own conclusion about what I’m studying.	3.69 ± 0.06	i.	Tracking down information for myself.	3.14 ± 0.07						
10.	When I’ve been communicating ideas, I’ve thought over how well I’ve got my points across	4.04 ± 0.06	j.	Information technology/computing skills (e.g., www, email, word processing).	3.59 ± 0.08						
11.	I’ve organised my study time carefully to make the best use of it.	3.47 ± 0.08									
12.	It has been important for me to follow the argument, or to see the reasons behind things.	3.87 ± 0.06									
13.	I’ve tended to take what we’ve been taught at face value without questioning it much.	2.91 ± 0.09*									
14.	I’ve tried to find better ways of tracking down relevant information in this subject	3.9 ± 0.06									
15.	Concentration has not usually been a problem for me, unless I’ve been really tired.	3.47 ± 0.09									
16.	In reading for this course unit, I’ve tried to find out for myself exactly what the author means.	3.73 ± 0.06									
17.	I’ve just been going through the motions of studying without seeing where I’m going.	2.99 ± 0.08*									
18.	If I’ve not understood things well enough when studying, I’ve tried a different approach.	3.88 ± 0.06									

SE, standers error of mean

*Mean scores < 3 points

**Table 4:** Section 2 Experiences of teaching and learning: mean score for each statement

2.1 Organization and structure			2.2 Teaching and learning			2.3 Students and teachers			2.4 Assessments and other set work		
#	**Statement**	**Mean score ± SE**	**#**	**Statement**	**Mean score ± SE**	**#**	**Statement**	**Mean score ± SE**	**#**	**Statement**	**Mean score ± SE**
1.	It was clear to me what I was supposed to learn in this course unit.	3.65 ± 0.07	7.	We were encouraged to look for links between this unit and others.	3.35 ± 0.09	21.	Students supported each other and tried to give help when it was needed.	3.86 ± 0.08	31.	It was clear to me what was expected in the assessed work for this course unit.	3.63 ± 0.08
2.	The topics seemed to follow each other in a way that made sense to me.	3.63 ± 0.08	8.	I can imagine myself working in the subject area covered by this unit.	3.42 ± 0.09	22.	I found most of what I learned in this course unit really interesting.	3.93 ± 0.07	32.	I was encouraged to think about how best to tackle the set work.	3.53 ± 0.08
3.	We were given a good deal of choice over how we went about learning.	3.31 ± 0.09	9.	The handouts and other materials we were given helped me to understand the unit.	3.74 ± 0.08	23.	Staff tried to share their enthusiasm about the subject with us.	3.55 ± 0.09	33.	I could see how the set work fitted in with what we were supposed to learn.	3.57 ± 0.08
4.	The course unit was well organised and ran smoothly.	3.34 ± 0.09	10.	On this unit, I was prompted to think about how well I was learning and how I might improve.	3.55 ± 0.08	24.	Talking with other students helped me to develop my understanding.	3.9 ± 0.07	34.	You had really to understand the subject to get good marks in this course unit.	3.82 ± 0.08
5.	We were allowed some choice over what aspects of the subject to concentrate on.	3.16 ± 0.08	11.	I could see the relevance of most of what we were taught in this unit.	3.85 ± 0.08	25.	Staff were patient in explaining things which seemed difficult to grasp.	3.59 ± 0.09	35.	The feedback given on my work helped me to improve my ways of learning and studying.	3.55 ± 0.08
6.	What we were taught seemed to match what we were supposed to learn.	3.79 ± 0.07	12.	We weren’t just given information; staff explained how knowledge is developed in this subject.	3.45 ± 0.08	26.	I enjoyed being involved in this course unit.	3.74 ± 0.08	36.	Doing the set work helped me to think about how evidence is used in this subject.	3.58 ± 0.07
			13.	The teaching encouraged me to rethink my understanding of some aspects of the subject.	3.72 ± 0.07	27.	Students’ views were valued in this course unit.	3.7 ± 0.07	37.	Staff gave me the support I needed to help me complete the set work for this course unit.	3.33 ± 0.08
			14.	The different types of teaching (lectures, tutorials, labs, etc.) supported each other well.	3.67 ± 0.08	28.	Staff helped us to see how you are supposed to think and reach conclusions in this subject.	3.45 ± 0.09	38.	To do well in this course unit, you had to think critically about the topics.	3.45 ± 0.07
			15.	Plenty of examples and illustrations were given to help us to grasp things better.	3.46 ± 0.08	29.	I found I could generally work comfortably with other students on this unit.	3.78 ± 0.07	39.	The set work helped me to make connections to my existing knowledge or experience.	3.68 ± 0.07
			16.	This unit has given me a sense of what goes on ‘behind the scenes’ in this subject area.	3.58 ± 0.08	30.	This course unit provided plenty of opportunities for me to discuss important ideas.	3.63 ± 0.08	40.	The feedback given on my set work helped to clarify things I hadn’t fully understood.	3.41 ± 0.08
			17.	The teaching in this unit helped me to think about the evidence underpinning different views.	3.5 ± 0.07						
			18.	How this unit was taught fitted in well with what we were supposed to learn.	3.64 ± 0.07						
			19.	This unit encouraged me to relate what I learned to issues in the wider world.	3.45 ± 0.08						
			20.	The web pages provided by staff helped me to understand the topics better.	3.5 ± 0.08						

SE, standers error of mean

*Mean scores < 3 points

In Section two “experiences of teaching and learning” the mean score was 3.57 ± 0.05, with no statements scored below three, and statement number 34 had the highest score of 3.82 ± 0.08 (Table 4).

Section three “Demands made by the course unit” had a mean score of 3.08 ± 0.05, with one statement “j” scored the highest mean of 3.59 ± 0.08 (Table 3). Two statements “c” and “e” scored below three, notably the statement “c” which refers to “the amount of work I was expected to do” had the overall lowest score of 2.74 ± 0.07,

Section four “what you learned from this course unit” had a mean score of 3.33 ± 0.05, in this section there was no difference observed between clinical and basic year students or between genders (Table 1). No statement scored below three in this section (Table 4).

**Fig. 1:**
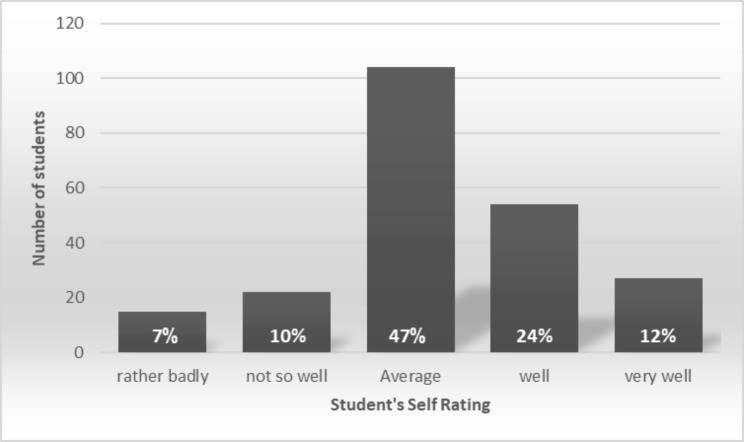
Section 5: How well do students think they are doing in the current module

According to section five, a one-statement section, more than one-third of students (35.88%) believe that they are performing above average in their current module (Fig. 1).

**Fig. 2:**
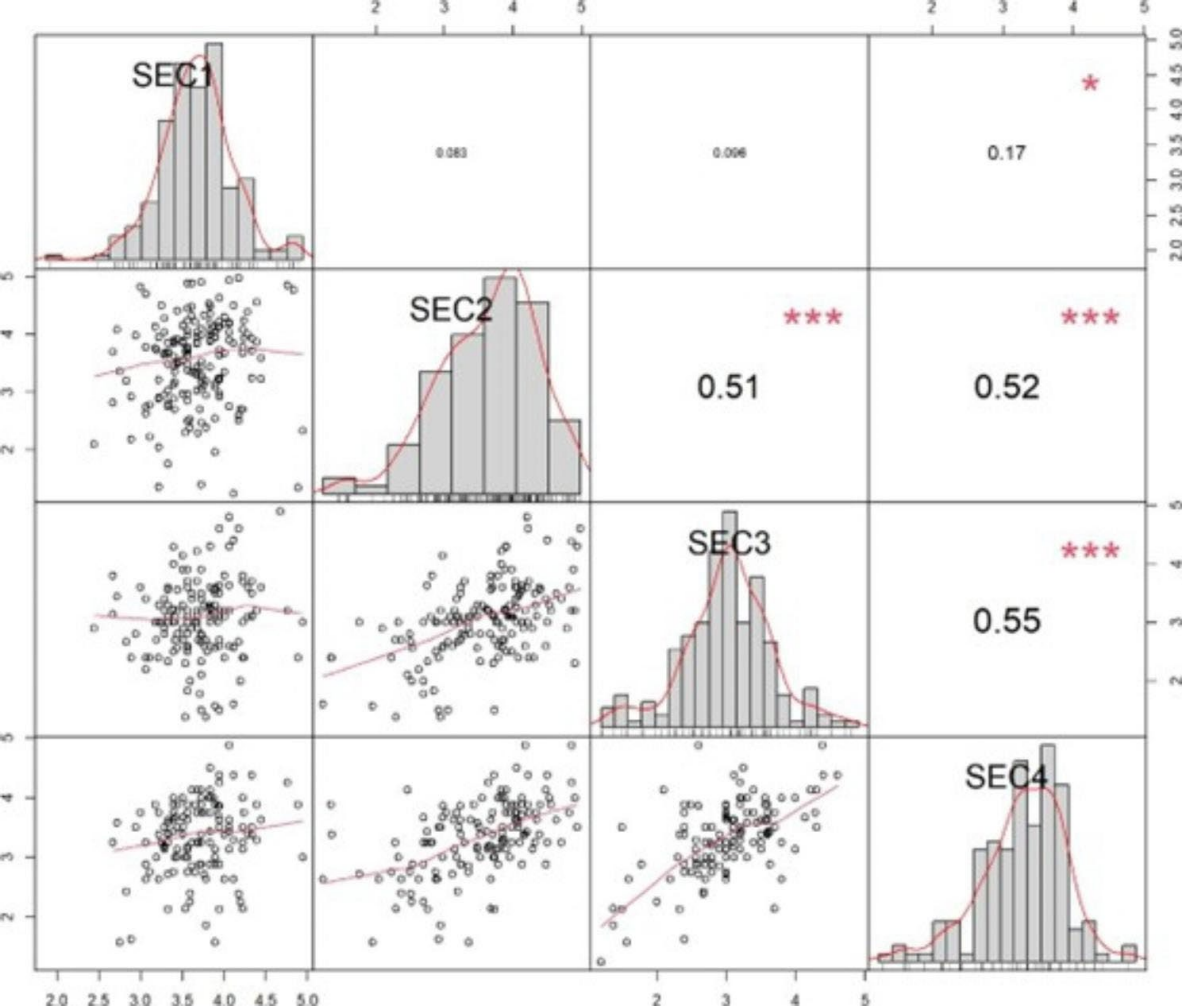
Correlation coefficient for the relationship between various teaching and learning sections

There was a strong association between all sections, as the correlation coefficient (*r*) was positive for the relationship between all sections. Section one “approaches to learning and studying” has the weakest correlation with other sections. while the correlation coefficients between the other three sections ranged from *r* = 0.51 to *r* = 0.55 (Fig. 2).

## Discussion

This preliminary assessment revealed an overall satisfactory perception of students about the TLE in our college. Senior students in clinical years have more acceptance of the learning environment compared to their counterparts. Students appreciated their approach to studying, experience of teaching and learning, and things learned from course units. However, they barely accepted the demand required in the process. Only 1/6th of students self-rated themselves as they are doing below average.

According to eight assessments done in universities in the region [23–27], only two assessments revealed students were positive toward the TLE [23, 27], interestingly, both assessments compared integrated to discipline-based curriculum and students were more positive toward the integrated curriculum in both assessments. The students perceive the TLE negatively in the discipline-based curriculum in both studies along with the remaining six assessments [23–27], moreover, three of these assessments in three different universities achieved low scores indicating “plenty of problems” according to the DREEM [28] tool [24–27].

The college has invested a substantial effort in designing its hybrid integrated curriculum and aligning it with the SaudiMED framework and the Saudi Vision 2030. The fact that this is a newly established college, with relatively limited material and human resources, situated in a small town with modest medical infrastructure leads us to suggest that this pleasing finding is mainly attributed to the own developed curriculum adopted by the college from day one. Integrated approach curriculum is associated with more appreciation [29–31]. Switching to a new integrated curriculum from a discipline-based one using diverse learning strategies was welcomed by almost all medical students from public Saudi, Malaysian and Caribbean universities, revealing a full agreement that integration between clinical and basic knowledge, and linking between different disciplines facilitate understanding of subjects [29, 30]. In a Caribbean medical school integrated curriculum helped students to review topics from different aspects and trained them for the license exams [31]. Conversely, only 60% were satisfied with their training and more than 50% complained of lacking clinical knowledge in basic science years in a school that adopted a non-integrated nonhybrid curriculum [32]. This finding might provide insight for newly established facilities on the reflection of curriculum design on the perception of the environment by students.

Unlike other sections, the respondents showed dissatisfaction with the demands made by the course unit (Sect. 3), particularly in demands required for acquiring knowledge and subject-based skills which clearly highlights an area for improvement.

The overall score showed that students in clinical years are more pleased with the TLE compared to their counterparts in basic science years. Studies showed that graduate and senior students tend to appreciate more their learning experience [32–34]. A systematic review found that in a curriculum that runs interactive methods of teaching students initially experience anxiety and unsatisfying feeling. Later on, those feelings tend to be positive [33]. The practical nature of the curriculum in senior years could easily explain this, another explanation related to maturity and passing early challenges [34].

The overall and sections mean scores did not show any difference between males and females. It is worth noting that females and males are studying in separate campuses, however, this does not affect their perception. Since the curriculum is unified between the two campuses this might support our previous interpretation of the expected role of the curriculum in our college. This finding is comparable to studies done in India in a newly established dental college in 2008 and in Saudi Arabia in 2013. However, some studies have found gender differences in the perception of TLE, with females having a more positive perception in some cases and a less positive perception in others. For instance, a study conducted in Saudi Arabia published in 2017 found that female students perceived the TLE better than their male counterparts, [35] while a study conducted in Dundee in 2004 found that female students were less satisfied with the TLE [24, 36].

The positive correlation revealed by this preliminary assessment between various aspects of the educational environment sends a convenient signal toward the balanced performance and state of harmony between the different parties of the TLE involved in the educational process.

### Areas for improvement

This assessment might direct future efforts in the College of Medicine in Majmaah toward (a) providing students with opportunities to learn organized studying and effort management, (b) empowering tutors to promote students’ capabilities in communicating knowledge and ideas, (c) allowing enough exposure to technologies to acquire minimum skills needed, (d) assessing and enhancing TLE during basic science years, and (e) conduct per module assessments to explore current results in depth. Moreover, fostering the role of central entities at the university level may be necessary to effectively address the identified areas for improvement and thoroughly investigate them within the university context.

### Strength and limitations

This work exhibits several strengths, such as being the first TLE assessment in our institution and a high response rate. It also highlights key gaps in the TLE and demonstrates the usefulness of ETLQs compared to the commonly used DREEM in similar studies in Saudi Arabia. However, one potential limitation of the study is that the ETLQ was designed to assess the environment at a course level, this could be a drawback for this assessment, however, experts suggested that it is a reliable instrument for use at degree level [37]. Additionally, there are limited published assessments using the ETLQ, which hinders comparisons. Furthermore, the study was conducted as a preliminary assessment with no previous assessments to compare with, and a small proportion of responses were received during the COVID-19 lockdown period, which may have affected the outcomes. Finally, the gender representation may not exactly match the gender ratio of enrolled students, we do not anticipate that this will have an impact on the findings.

Future assessments might consider implementing measures such as providing customised links to the questionnaire, ensuring a single response per student, and validating the GPA to further improve the validity and reliability of study outcomes.

## Conclusion

This assessment revealed that students at the College had a positive perception of the TLE. However, it was observed that students face challenges in coping with the demands of acquiring knowledge and subject-based skills, as well as in appreciating the TLE during their basic science years. These findings highlight the need for an atmosphere that allows students to meet the required demands, and develop a greater appreciation for the TLE especially during basic science years. Further qualitative assessment such as focus group is required to understand the challenges students go through, and the support they need, and. Overall, addressing these issues is essential to gain a comprehensive understanding of the TLE in the College of Medicine in Majmaah.

## Data Availability

The datasets used and analysed during the current study are available from the corresponding author on reasonable request.
